# Determinants of the type of health care sought for symptoms of Acute respiratory infection in children: analysis of Ghana demographic and health surveys

**DOI:** 10.1186/s12887-021-02990-9

**Published:** 2021-11-17

**Authors:** Leslie Danquah, Prince Michael Amegbor, Dawit Getnet Ayele

**Affiliations:** 1 Department of Geographic Science, School of Geosciences, University of Energy and Natural Resources, Sunyani, Ghana; 2 Department of Environmental Science, Aarhus University, Roskilde, Denmark; 3 Institute of Human Virology, School of Medicine, University of Maryland, Baltimore, USA

**Keywords:** Acute respiratory infection (ARI), Ghana, Demographic and Health Survey (GDHS), Care-seeking behavior, Bayesian spatial analysis

## Abstract

**Background:**

Globally, acute respiratory infection (ARI) is a leading cause of infant and childhood morbidity and mortality. Currently, it is estimated that 50 million cases of childhood ARI are untreated. In this study, we identified determinants of the type of treatment sought for symptoms of childhood acute respiratory infection (ARI), including non-treatment, amongst a nationally representative sample of children under five years in Ghana.

**Methods:**

In total, 1 544 children were studied by a secondary analysis of pooled survey data from the 1993, 1998, 2003, 2008, and 2014 Ghana Demographic and Health Surveys (GDHS). Cross-tabulations, chi-square, multinomial logistic regression, and Bayesian hierarchical spatial logistic regression analyses were used to identify relationships between the type of treatment sought and maternal socio-economic and household characteristics.

**Results:**

Seeking medical care was significantly associated with child age (RRR= 1.928, 95 % CI 1.276 – 2.915), maternal employment status (RRR = 1.815, 95 % CI 1.202 – 2.740), maternal health insurance status, (RRR = 2.618, 95 % CI 1.801 – 3.989), children belonging to middle (RRR = 2.186, 95 % CI 1.473 – 3.243), richer (RRR = 1.908, 95 % CI 1.145 – 3.180) and richest households (RRR = 2.456, 95 % CI 1.363 – 4.424) and the 1998 survey period (RRR = 0.426, 95 % CI 0.240 – 7.58). Seeking self-care or visiting a traditional healer was significantly associated with maternal educational status (RRR = 0.000, 95 % CI 0.000 – 0.000), and the 1998 (RRR= 0.330, 95 % CI 0.142 – 0.765), 2003 (RRR= 0.195, 95 % CI 0.071 – 0.535), 2008 (RRR= 0.216, 95 % CI 0.068 – 0.685) and 2014 (RRR= 0.230, 95 % CI 0.081 – 0.657) GDHS periods. The probability that the odds ratio of using medical care exceeded 1 was higher for mothers/caregivers in the Western, Ashanti, Upper West, and Volta regions.

**Conclusions:**

Government policies that are aimed at encouraging medical care-seeking for children with ARI may yield positive results by focusing on improving maternal incomes, maternal NHIS enrolment, and maternal household characteristics. Improving maternal education could be a positive step towards addressing challenges with self-care or traditional healing amongst children with ARI.

## Background

Globally, acute respiratory infections (ARIs) mortality [1], with the annual untreated cases of childhood ARI estimated to be over 50 million [2]. Furthermore, it is estimated that 80 % of all ARI-related deaths among children under 5 years of age occur in developing countries, making it a leading cause of infant mortality in these countries [3]. Symptoms of ARIs include respiratory rate greater than or equal to 70 breaths per minute, severe chest wall retractions, cough and inability to feed and drink [4].

Children are particularly vulnerable to ARIs, because of their inability to adequately protect themselves from the associated environmental risks, their relatively immature immune systems and physical development [4]. Children under five years are not usually able to seek health care themselves; they must rely on adults for this. This responsibility usually falls to the mother or other female caregivers in the family [5]. With differing sociocultural roles in the home, males are often regarded as breadwinners whereas females are seen as homemakers, which includes taking care of the children [5].

Securing child health depends on appropriate health care-seeking. Insight from existing studies indicates that such behaviour is associated with a variety of factors including maternal education, maternal age, household size, maternal ethnicity, and household socio-economic status [6–9]. Distance from health care facilities is known to play a key role. For instance, a study in rural Tanzania found that mothers who lived one or more kilometers from a health center were less likely to access health care [8]. Mothers were also more likely to seek health care for children aged 24 months or younger [8, 10]. Some studies also suggest that mothers seldom seek health care for their child in the early stages of a child’s illness [6, 10]. This practice would have negative implications for child health. Some studies indicate that over 30 % of child deaths can be attributed to late care-seeking [11]. Similar findings have been reported in Ghana, where ARIs accounted for 20 % of all annual deaths among children under five years [12]. Recent studies have also shown an association between socio-demographic and cultural factors such as distance from facilities, income, ethnicity and household size and access to health insurance to be associated with maternal health care seeking in Ghana [13, 14]. Another study also showed that child mortality and health-seeking behavior were a function of social factors such as maternal education, place of residence, and family income [15].

The healthcare system in Ghana can be considered to be pluralistic in that it is characterized by two parallel systems: the Traditional and the Orthodox (or medical) systems [16–20]. The traditional system is the oldest and most widely used care system due to its accessibility, acceptability, affordability, and availability [16, 17]. It includes the use of indigenous knowledge, spiritual therapies, herbal medicines, manual techniques, and in some cases, modern medical equipment to diagnose and treat ailments [20–22]. Traditional medical practice in Ghana is regulated by the Traditional Medical Practice Council which gains its mandate from the Traditional Medical Practice Act 575, 2000 [23]. Despite the efforts by the council, unlicensed practitioners and others who practice in secrecy hinder the proper regulation of traditional medical practice [24]. The use of traditional medicine is more widespread in rural rather than urban areas, partially due to the skewed availability of modern health care facilities [25, 26] but mainly due to cultural norms, the desire to be part of the healing process, perceived displeasure with the medicalization of western medicine, and perceptions on the quality of care [27].

Orthodox medicine, by contrast is characterized by the use of scientific methods and principles to arrive at a diagnoses and treatment of diseases [18, 21, 22]. In Ghana, adherents to orthodox medicine, seek care in health care facilities that are either private or publicly owned. These include hospitals, clinics, polyclinics, pharmacies, health centers, and Community-Based Health Planning and Services (CHPS) compounds. The inequitable spatial distribution of personnel and resources for orthodox medicine and corresponding access to these facilities has been reported [20]. A study in 2011 suggested that, overall, whereas the ratio of traditional medical practitioners to human population stood at 1:200, orthodox doctor–population ratio stood at 1: 20,000 [28].

Studies on maternal health care seeking for childhood ARI, especially in the Ghanaian context, are very limited. Existing studies have mainly focused on the determinants of ARI among children, with limited or no focus on care-seeking behavior among the mothers [29–31]. The findings of these studies show that maternal and household socioeconomic factors are significantly associated with the ARI symptoms among children under age five. There is also a limited knowledge on the temporal trend in care-seeking for ARI, given that policies such as the Child Health Policy 2007–2015, Community-based Health Planning and Services (CHPS) policy, and Free Maternal Health Policy, have been introduced in the healthcare sector in the last three decades [30–33]. To the best of our knowledge, studies on maternal health care seeking for childhood ARI have not considered a recent nationally representative survey of children in Ghana. We, therefore, contribute to this growing body of literature by reporting on the determinants of care-seeking for childhood ARI using data from five successive National Demographic and Health Surveys conducted in Ghana.

Knowledge of the determinants would be useful for planning interventions that could help improve health care-seeking and ultimately secure child health. This is most important, given that ARIs are one of the leading causes of morbidity and mortality among children under five years in Ghana and internationally [1, 3, 12]. Additionally, such information would assist in making policy decisions on the attainment of Sustainable Development Goal 3; Good health and well-being. The objectives of the study are two-fold. First, the study aims to assess the association between socioeconomic factors and the type of treatment sought for ARI. Secondly, the study explores the specific effect of place of residence on seeking medical care for childhood ARI symptoms using a Bayesian hierarchical spatial logistic regression.

### Data and methods

The study used data from the following Ghana Demographic and Health Survey (GDHS) over the years: 1993, 1998, 2003, 2008, and 2014 [34–38]. The GDHS is a nationally representative survey of women aged 15 to 49 years and men aged 15 – 59 years. Its main objective is to capture information on fertility, maternal and child health as well as family planning and attitudes towards HIV/AIDS and other sexually transmitted infections [32]. Respondents for the surveys were selected through a two-stage sampling process. The design used 20 sampling strata from stratification of each of the ten administrative regions into urban and rural areas. The first stage of the sampling process involved selecting census enumeration areas (EAs) in each stratum. The probability of selection of each EA was proportional to the size of the EA – that is, the number of residential households in the EA. In the second stage, households were randomly selected within each EA, and all women (aged 15-49 years) who were members of the household or who had spent the night before the survey in the house were interviewed. The birth history and health information of children born to eligible women in the last five years before the survey were collected as part of the data. This information was kept in the child recode dataset – the data used for this study. Detailed descriptions of the GDHS surveys and the sampling methods are available in the final reports for the surveys [32–36]. The sample for this study was children under age five who were ill with acute respiratory infections (ARI). The samples of children under age five with ARI in the successive GDHS used in this study were for each survey 2 204 (GDHS 1993), 3 298 (GDHS 1998), 3 844 (GDHS 2003), 2 992 (GDHS 2008), and 5 884 (GDHS 2014).

### Measures

The outcome variable for this study was the type of treatment sought for children with ARI symptoms. In the GDHS, ARI among children was identified by asking mothers and caregivers of children under age five whether their children had been ill with a cough accompanied by short and rapid breathing in the two weeks before the survey. The mothers and caregivers were then asked if they had sought treatment for the ARI symptoms and where the treatment had been sought. For our outcome variable, children with ARI symptoms whose mothers or caregivers did not seek treatment were categorized into a single group and coded as “0 = No treatment sought”. We operationally defined seeking medical care as a mother or caregiver seeking an expert opinion or treatment from a public or private hospital or clinic, outside the home, for a child who shows symptoms of ARI. Therefore, children whose mothers or caregivers sought treatment in public health care facilities (such as a government hospital, government health center/clinic, government health post or CHPS) or at private health care facilities (e.g. private hospital, private clinic, private doctors, mobile clinic and maternity home), but excluding a pharmacy or drug store, were classified into a single group and coded as “1 = Sought medical care”. Children whose mothers or caregivers sought other sources of treatment, such as ‘pharmacy or drug store’, ‘traditional healer’ and ‘drug peddler’, were classified and coded as “2 = Self-care or sought traditional treatment”.

The independent variables were child demographic characteristics, maternal socioeconomic status, household characteristics, and place of residence. The age and sex of the child were included as child demographic characteristics. Maternal socioeconomic and demographic characteristics were age, marital status, religion, education, employment, and health insurance coverage. Maternal education was grouped into four categories and coded as: “0 = No formal education”, “1 = Primary education”, “2 = Secondary or high school education”, and “3 = Higher education”. The responses for maternal employment status were derived from the GDHS question that asks respondents whether they had been employed during the 12 months prior to the survey. Respondents who indicated they had worked in the past year, were currently working and those with employment, but on leave were grouped into a single category and coded as “1 = Employed”; while those who indicated they had not worked in the past year were coded as “0 = Unemployed”. Another maternal characteristic captured was health insurance status – that is, whether the mother or caregiver was covered by health insurance – with a binary variable coded as “0 = Uninsured” and “1 = Insured”.

Household characteristics used as possible predictors of type of treatment sought for ARI symptoms were: household wealth index (a DHS construct based on assets ownership and housing characteristics of each household) and sex of household head. Households were classified as poorest, poorer, middle, richer, and richest under the household wealth index. Place of residence was also included as an independent variable; it was categorized and coded as “0 = urban” or “1 = rural”. Finally, a variable capturing the GDHS periods or years was also included as an independent variable, with the 1993 GDHS as the reference category.

### Analysis

Univariate, bivariate, and multivariate analyses were performed concerning the objectives of this study. We first conducted a cross-tabulation analysis to examine the distribution of the study sample characteristics by type of treatment sought for children with ARI symptoms. We performed a Pearson Chi-square test of independence to identify any association between the predictor variables and the type of treatment sought for children with ARI symptoms; while the Cramer’s V test was used to determine the strength of the association. Next, we conducted a multinomial logistic regression analysis to examine the effect of maternal socioeconomic status and household characteristics on the type of treatment sought for children with ARI symptoms among children under 5 years of age. The outcome variable of this study was a nominal variable with three categories hence the choice of multinomial logistic regression modeling. To facilitate easy interpretation of the result, we report the relative risk ratio (RRR) of the multinomial logistic regression model – which is the ratio of the probability of choosing medical treatment and self-care or using a traditional healer over the probability of not seeking treatment (the baseline category). The cross-tabulation and multinomial logistic regression were conducted using STATA version 16 [37]. We controlled for the survey design effects using the ‘svyset’ command in STATA to adjust for the sampling clusters and weights. We also estimated the design effect to provide insight into the efficiency of the sample used in this study. The first design effect – Deff - is a ratio of the variance estimate from our sample and the variance estimate from a hypothetical sample of the same size drawn as simple random sampling (SRS). A Deff value greater than 1 implies the study sample is more efficient than SRS. The second design effect estimate – Deft – is the ratio of the standard errors in the study sample and in the SRS.

The final analysis was a Bayesian hierarchical spatial logistic regression to account for potential spatial dependence among the administrative regions used in the sampling process. The sampling method used in the GDHS means the data from the surveys are geographically distributed data. Due to the sampling approach, traditional regression models such as the multinomial logistic regression model used in the previous analysis do not account for potential spatial dependence. The concept of spatial dependency is based on Tobler’s First Law of Geography, which states, “everything is related to everything else, but near things are more related than distant things” [38]. This concept violates the independently distributed observations and error assumptions of regression models. To address this issue, we included a spatial random effect term in our Bayesian model to account for potential spatial dependency between the administrative regions. In the modeling, we assume a Besag-York-Mollie (BYM) specification. BYM specification proposed by accounts for both smoothened spatial structure of the data based on the concept of spatial dependency (spatial autocorrelation) and an unstructured spatial effect. The Unstructured spatial effect is based on the assumptions that the effect of the spatial units or administrative regions may be independent of neighboring units or regions – independent region-specific noise (unstructured spatial effect) [39]. The BYM modeling specification, thus addresses the issue of potential bias where there is no spatial dependency [39]. For the Bayesian hierarchical spatial logistic regression, we created a dummy variable out of the original outcome variable and coded it as “1 = sought medical treatment” and “0 = did not seek treatment or self-care or sough traditional healer”.

The Bayesian hierarchical spatial logistic regression was implemented in the open-access R software [40] using the R-INLA package [39–43]. We visualized the results for the region-specific odds ratio of using a medical treatment for ARI and the posterior probability that the odds ratio of using a medical treatment for ARI symptoms exceeds one (exceedance probability) in R software using the ‘tmap’ and ‘tmaptools’ packages [41].

## Results

### Descriptive characteristics

Table 1 shows the summary statistics of the study variables. From the pooled data, 1,544 children (8.47 %) had ARI symptoms out of the total 18,222 children aged under 5 years. Most mothers or caregivers (hereinafter called mother) (57.32 %) did not seek treatment for children with ARI symptoms; while 38.41 % of mothers sought medical care, only 4.27 % had used self-care or consulted a traditional healer, respectively. Table 1 also indicates that slightly over half (51.04 %) of children with ARI were males. Most mothers of children under age 5 with ARI symptoms were married (74.61 %), Christian (71.76 %), uneducated (41.65 %), lived in the poorest and poorer household (55.76 %), and rural areas (72.93 %). Over 50 % of mothers were less than 30 years old. Moreover, 75 % of mothers were uninsured with NHIS with most living in male-headed households (74.81 %). Over 50 % of children were 2 years of age or younger. The majority of children with ARI symptoms also lived in a male-headed household (74.81). Among the different regions, the northern region contributed the most cases of ARI symptoms, accounting for 15.54 % of total cases.

**Table 1:** Descriptive summary of study variables (n = 1,544)

	Frequency	Percentage
**Treatment for ARI symptoms**		
No treatment sought	885	57.32
Medical treatment	593	38.41
Self-care or traditional healer	66	4.27
**Child Age**		
*4 years*	193	12.50
*3 years*	235	15.22
*2 years*	314	20.34
*1 year*	429	27.78
*less than 1 year*	373	24.16
**Child Sex**		
*Male*	788	51.04
*Female*	756	48.96
**Maternal Age**		
*15-19 years*	67	4.34
*20-24 years*	328	21.24
*25-29 years*	430	27.85
*30-34 years*	321	20.79
*35-39 years*	231	14.96
*40-44 years*	123	7.97
*45-49 years*	44	2.85
**Marital Status - Mother**		
*Married*	1152	74.61
*Common-law*	224	14.51
*Widowed/divorced/separated*	106	6.87
*Never-in-union*	62	4.02
**Religion - Mother**		
*Christian*	1108	71.76
*Islam*	234	15.16
*Traditional*	86	5.57
*Other*	92	5.96
*No religion*	24	1.55
**Highest level of Education - Mother**		
*No formal education*	643	41.65
*Primary*	370	23.96
*Secondary or High School*	497	32.19
*Post-secondary*	34	2.20
**Employment status - Mother**		
*Unemployed*	290	13.54
*Employed*	1335	86.46
**Health Insurance status**		
*Uninsured*	1170	75.78
*Insured*	374	24.22
**Sex of Household head**		
*Male*	1155	74.81
*Female*	389	24.19
**Household wealth**		
*Poorest*	519	33.61
*Poorer*	342	22.15
*Middle*	297	19.24
*Rich*	224	14.51
*Richest*	162	10.49
**Region of Residence**		
Western	143	9.26
Central	118	7.64
Greater Accra	115	7.54
Volta	170	11.01
Eastern	160	10.36
Ashanti	187	12.11
Brong-Ahafo	132	8.55
Northern	240	15.54
Upper East	119	7.71
Upper West	160	10.36
**Place of residence**		
*Urban*	418	27.07
*Rural*	1126	72.93
**DHS Period**		
*1993 GDHS*	98	6.35
*1998 GDHS*	429	27.78
*2003 GDHS*	350	22.67
*2008 GHDS*	303	19.62
*2014 GDHS*	364	23.58

Table 2 presents the results of the cross-tabulation analysis, Chi-square test, and Cramer’s V test. The chi-square test of independence shows that region of residence, rural/urban residence, and the maternal characteristics (age, religion, education, employment, health insurance coverage), household wealth and GDHS period were significantly associated with ARI symptoms (p < 0.05). The Cramer’s V test reveals maternal health insurance status was moderately associated with the type of treatment sought for children’s ARI symptoms (V>=0.20). The remaining independent variables were weakly associated with the type of treatment sought for children’s ARI symptoms (V < 0.20). The cross-tabulation distribution shows that the Volta region had the highest proportion (71.18 %) of mothers who did not seek any form of treatment for children with ARI symptoms. On the other hand, the regions with highest proportion of mothers seeking medical treatment for children with ARI symptoms were Upper West (48.75 %) and Upper East (45.38 %) regions. The result shows that mothers did not seek care for approximately 62 % of children with ARI symptoms living in rural areas; while medical care was sought for 52 % of children with ARI symptoms in urban areas. Most mothers who followed traditional religion (73.26 %), had no formal education (61.12 %), only primary education (59.46 %), and secondary education (52.31 %), while the majority of mothers with post-secondary education (64.71 %) sought medical care for children with ARI symptoms. For children with ARI symptoms living with mothers without health insurance cover, 61.71 % received no treatment compared to 32.99 % and that had received medical care and 5.30 % who received self-care or care from a traditional healer. Of the children living with mothers with health insurance cover, 55.35 % had received medical care. Also, the majority of children with ARI symptoms did not receive treatment if they lived in the poorest (64.93 %), poorer (61.99 %), or middle (54.55 %) wealth indexes households did not receive treatment; while 56.17 % of children in the richest households received medical care.

**Table 2:** Maternal and household socioeconomic characteristics by type of treatment sought for ARI symptoms (n = 1,544)

	No Treatment		Medical care		Self-care or Traditional healer		Chi-Square Test (Cramer’s V Test)
	Frequency	Percentage	Frequency	Percentage	Frequency	Percentage	
**Region of Residence**							
Western	76	53.15	62	43.36	5	3.50	40.507 (0.115)**
Central	79	66.95	32	27.12	7	5.93	
Greater Accra	57	49.57	51	44.35	7	6.09	
Volta	121	71.18	45	26.47	4	2.35	
Eastern	93	58.13	59	36.88	8	5.00	
Ashanti	102	54.55	76	40.64	9	4.81	
Brong-Ahafo	72	54.55	55	41.67	5	3.79	
Northern	145	60.42	81	33.75	14	5.83	
Upper East	63	52.94	54	45.38	2	1.68	
Upper West	77	48.13	78	48.75	5	3.13	
**Child Age**							
*4 years*	118	61.14	64	33.16	11	5.70	14.533 (0.068)
*3 years*	146	62.13	78	33.19	11	4.68	
*2 years*	183	58.28	117	37.26	14	4.46	
*1 year*	221	51.52	194	45.22	14	3.26	
*less than 1 year*	217	58.18	140	37.53	16	4.29	
**Child Sex**							
*Male*	437	55.46	313	39.72	38	4.82	2.826 (0.043)
*Female*	448	59.26	280	37.04	28	3.70	
**Place of residence**							
*Urban*	189	45.22	218	52.15	11	2.63	46.469 (0.174)***
*Rural*	696	61.81	375	33.30	55	4.88	
**Maternal Age**							
*15-19 years*	41	61.19	21	31.34	5	7.46	22.715 (0.086)*
*20-24 years*	203	61.89	116	35.37	9	2.74	
*25-29 years*	234	54.42	178	41.40	18	4.19	
*30-34 years*	182	56.70	120	37.38	19	5.92	
*35-39 years*	119	51.52	102	44.16	10	4.33	
*40-44 years*	77	62.60	45	36.59	1	0.81	
*45-49 years*	29	65.91	11	25.0	4	9.09	
**Marital Status - Mother**							
*Married*	651	56.51	456	39.58	45	3.91	9.545 (0.056)
*Common-law*	135	60.27	79	35.27	10	4.46	
*Widowed/divorced/separated*	68	64.15	30	28.30	8	7.55	
*Never-in-union*	31	50.00	28	45.16	3	4.84	
**Religion - Mother**							
*Christian*	610	55.05	453	40.88	45	4.06	25.012 (0.090)**
*Islam*	138	58.97	89	38.03	7	2.99	
*Traditional*	63	73.26	16	18.60	7	8.14	
*Other*	61	66.30	25	27.17	6	6.52	
*No religion*	13	54.17	10	41.67	1	4.17	
**Highest level of Education - Mother**							
*No formal education*	393	61.12	221	34.37	29	4.51	22.464 (0.085)***
*Primary*	220	59.46	132	35.68	18	4.86	
*Secondary or High School*	260	52.31	218	43.86	19	3.82	
*Higher education*	12	35.29	22	64.71	0	0.00	
**Employment status - Mother**							
*Unemployed*	127	66.49	61	31.94	3	1.57	8.037 (0.075)*
*Employed*	710	56.57	493	39.28	52	4.14	
**Health Insurance status**							
*Uninsured*	722	61.71	386	32.99	62	5.30	64.987 (0.205)***
*Insured*	163	43.58	207	55.35	4	1.07	
**Sex of Household head**							
*Male*	654	56.62	453	39.22	48	4.16	1.329 (0.029)
*Female*	231	59.38	140	35.99	18	4.63	
**Household wealth**							
*Poorest*	337	64.93	152	29.29	30	5.78	58.270 (0.137)***
*Poorer*	212	61.99	114	33.33	16	4.68	
*Middle*	162	54.55	127	42.76	8	2.69	
*Rich*	107	47.77	109	48.66	8	3.57	
*Richest*	67	41.36	91	56.17	4	2.47	
**DHS Period**							
*1993 GDHS*	48	48.98	39	39.80	11	11.22	63.556 (0.144)***
*1998 GDHS*	296	69.00	107	24.94	26	6.06	
*2003 GDHS*	189	54.00	149	42.57	12	3.43	
*2008 GHDS*	164	54.13	130	42.90	9	2.97	
*2014 GDHS*	188	51.65	168	46.15	8	2.20	
***p < 0.001, **p < 0.01, *p < 0.05							

The results in Table 3 for the multinomial logistic regression model shows that child age, maternal employment status, maternal health insurance status, household wealth, and GDHS period are significantly associated with choosing medical care over not seeking treatment. Only maternal education and GDHS period were significant predictors of choosing self-care or seeking a traditional healer over not seeking treatment for ARI symptoms among children aged under 5 years. Mothers of children aged 1 year old were 93 % (RRR=1.926, p < 0.01) more likely to use medical treatment for ARI symptoms over not seeking treatment. The relative risk ratio for choosing medical care over not seeking treatment was 1.815 times (p < 0.01) greater among employed mothers of children with ARI symptoms than unemployed mothers, adjusting for other variables in the model. Likewise, insured mothers of children with ARI symptoms had a greater relative risk ratio for choosing medical care over not seeking treatment (RRR=2.681, p < 0.001). Children with ARI symptoms living in middle, richer and richest households had a greater relative risk ratio of using medical care compared to those living in the poorest households. The relative risk ratio for choosing medical care was 0.426 times (p < 0.01) lower among children in the 1998 GDHS, compared to those in the 1993 GDHS. The relative risk ratio for self-care or seeking a traditional healer over not seeking treatment was greater among mothers with higher education relative to that among uneducated mothers. However, the magnitude was less negligible (RRR=0.000, p < 0.000). The relative risk ratio for using self-care or seeking a traditional healer was lower among children in all the GDHS survey periods compared to the reference GDHS year (1993 GDHS). Except for participants who did not belong to any religious denomination and participants from the 2014 GDHS, the design effect estimates (DEFF) show that the sample for all response categories was more efficient than SRS.

**Table 3:** Multinomial logistic regression analysis of determinants of treatment sought for ARI symptoms

	Medical Care		Design Effect		Self-Care or Traditional healer		Design Effect	
	RRR (95 % CI )	P-value	DEFF	DEFT	RRR (95 % CI )	P-value	DEFF	DEFT
**Child Age**								
*4 years (ref)*								
*3 years*	0.998 (0.621 -1.602)	0.992	1.166	1.079	0.709 (0.283 - 1.778)	0.463	1.094	1.046
*2 years*	1.498 (0.952 - 2.359)	0.081	1.209	1.099	0.720 (0.282 - 1.835)	0.490	1.160	1.077
*1 year*	1.928 (1.276 - 2.915)**	0.002	1.108	1.053	0.486 (0.179 - 1.319)	0.157	1.273	1.128
*less than 1 year*	1.520 (0.973 - 2.372)	0.066	1.171	1.082	0.527 (0.226 - 1.225)	0.137	1.047	1.023
**Child Sex**								
*Male (ref)*								
*Female*	0.967 (0.748 - 1.249)	0.800	1.261	1.123	0.726 (0.421 - 1.254)	0.251	1.091	1.045
**Place of residence**								
*Urban (ref)*								
*Rural*	0.840 (0.577 - 1.223)	0.362	1.366	1.169	1.346 (0.597 - 3.037)	0.474	1.223	1.106
**Maternal Age**								
*15-19 years (ref)*								
*20-24 years*	1.063 (0.565 - 2.001)	0.849	1.125	1.061	0.583 (0.110 - 3.078)	0.524	1.632	1.277
*25-29 years*	1.308 (0.677 - 2.525)	0.423	1.179	1.086	0.839 (0.153 - 4.577)	0.839	1.703	1.305
*30-34 years*	1.037 (0.513 - 2.092)	0.920	1.253	1.119	1.171 (0.217 - 6.309)	0.854	1.656	1.287
*35-39 years*	1.414 (0.706 - 2.833)	0.328	1.156	1.075	0.824 (0.129 - 5.255)	0.838	1.689	1.299
*40-44 years*	1.404 (0.659 - 2.991)	0.378	1.149	1.072	0.164 (0.211 - 2.046)	0.160	1.395	1.181
*45-49 years*	0.768 (0.299 - 1.977)	0.583	1.147	1.071	1.499 (0.205 - 10.932)	0.690	1.434	1.198
**Marital Status - Mother**								
*Married (ref)*								
*Common-law*	0.777 (0.522 - 1.156)	0.211	1.360	1.660	2.542 (0.437 - 14.803)	0.527	1.412	1.188
*Widowed/divorced/separated*	0.636 (0.377 - 1.073)	0.090	1.284	1.133	1.335 (0.546 - 3.267)	0.137	1.261	1.123
*Never-in-union*	1.459 (0.726 - 2.933)	0.288	1.219	1.104	1.955 (0.808 - 4.727)	0.299	1.346	1.160
**Religion - Mother**								
*Christian (ref)*								
*Islam*	0.970 (0.643 - 1.461)	0.885	1.284	1.133	0.740 (0.278 - 1.970)	0.546	1.047	1.024
*Traditional*	0.539 (0.220 - 1.319)	0.176	0.952	0.976	1.830 (0.602 - 5.562)	0.286	1.086	1.042
*Other*	0.613 (0.320 - 1.175)	0.147	1.272	1.128	0.905 (0.318 - 2.572)	0.851	1.073	1.036
*No religion*	1.701 (0.511 - 5.667)	0.387	1.550	1.245	1.517 (0.165 - 13.953)	0.712	0.997	0.998
**Highest level of Education - Mother**								
*No formal education (ref)*								
*Primary*	0.956 (0.658 - 1.387)	0.815	1.399	1.183	1.191 (0.604 - 2.347)	0.614	1.085	1.042
*Secondary or High School*	1.051 (0.722 - 1.530)	0.792	1.424	1.193	1.390 (0.592 3.263)	0.449	1.367	1.169
*Higher education*	1.784 (0.549 - 5.801)	0.334	1.547	1.244	0.000 (0.000 - 0.000)***	0.000	1.421	1.192
**Employment status - Mother**								
*Unemployed (ref)*								
*Employed*	1.815 (1.202 - 2.740)**	0.005	1.397	1.161	2.159 (0.719 - 6.482)	0.170	1.162	1.078
**Health Insurance status**								
*Uninsured (ref)*								
*Insured*	2.681 (1.801 - 3.989)***	0.000	1.196	1.094	0.490 (0.142 - 1.686)	0.257	1.589	1.261
**Sex of Household head**								
*Male (ref)*								
*Female*	0.874 (0.628 - 1.217)	0.424	1.347	1.161	0.961 (0.502 - 1.838)	0.903	1.121	1.059
**Household wealth**								
*Poorest (ref)*								
*Poorer*	1.225 (0.839 - 1.789)	0.293	1.172	1.082	0.794 (0.363 - 1.631)	0.529	1.295	1.138
*Middle*	2.186 (1.473 - 3.243)***	0.000	1.246	1.116	0.532 (0.226 - 1.251)	0.148	1.173	1.083
*Richer*	1.908 (1.145 - 3.180)*	0.013	1.378	1.174	1.024 (0.377 - 2.785)	0.962	1.326	1.152
*Richest*	2.456 (1.363 - 4.424)**	0.003	1.264	1.124	0.701 (0.180 - 2.731)	0.609	1.367	1.169
**DHS Period**								
*1993 GDHS (ref)*								
*1998 GDHS*	0.426 (0.240 - 0.758)**	0.004	1.397	1.182	0.330 (0.142 - 0.765)**	0.010	1.061	1.030
*2003 GDHS*	0.973 (0.543 - 1.745)	0.933	1.274	1.129	0.195 (0.071 - 0.535)**	0.002	1.066	1.033
*2008 GHDS*	0.748 (0.394 - 1.423)	0.379	1.312	1.146	0.216 (0.068 - 0.685)**	0.009	1.069	1.034
*2014 GDHS*	0.509 (0.257 - 1.011)	0.054	1.267	1.126	0.230 (0.081 - 0.657)**	0.006	0.975	0.987
CI = Confidence Interval, ***p < 0.001, **p < 0.01, *p < 0.05								

Table 4 displays the results of the Bayesian hierarchical spatial logistic regression model. The results indicate mothers of children aged 1 year were more likely (MOR=1.790) to seek medical care for ARI symptoms, compared to those with children aged 4 years. Mothers practiced traditional religion were less likely to seek medical care for children with ARI symptoms, compared to Christian mothers. Children of employed and insured mothers, as well as those living in non-poorest households, were more likely to use medical care compared to not seeking treatment or self-care or using traditional treatment. The model also shows that 1 % of the variability in seeking medical over not seeking treatment or >self-care or using traditional treatment could be attributed to the mother’s region of residence.

**Table 4:** Bayesian Hierarchical Spatial Logistic regression analysis of determinants of using medical care for ARI symptoms

	Mean (SD)	MOR	95 % CI
Intercept	**-1.599 (0.523)**	**0∙202**	**-2.641 - -0.584**
**Child Age**			
*4 years (ref)*			
*3 years*	0.000 (0.222)	1∙000	-0.436 - 0.438
*2 years*	0.182 (0.21)	1∙200	-0.229 - 0.598
*1 year*	**0.582 (0.198)**	**1∙790**	**0.195 - 0.974**
*less than 1 year*	0.34 (0.206)	1∙405	-0.061 - 0.747
**Child Sex**			
*Male (ref)*			
*Female*	-0.122 (0.114)	0∙885	-0.346 - 0.1
**Place of residence**			
*Urban (ref)*			
*Rural*	-0.324 (0.168)	0∙723	-0.654 - 0.005
**Maternal Age**			
*15-19 years (ref)*			
*20-24 years*	0.246 (0.318)	1∙279	-0.367 - 0.883
*25-29 years*	0.412 (0.321)	1∙510	-0.206 - 1.055
*30-34 years*	0.187 (0.332)	1∙206	-0.453 - 0.850
*35-39 years*	0.502 (0.342)	1∙652	-0.157 - 1.184
*40-44 years*	0.451 (0.37)	1∙570	-0.268 - 1.186
*45-49 years*	-0.352 (0.491)	0∙703	-1.330 - 0.599
**Marital Status - Mother**			
*Married (ref)*			
*Common-law*	-0.198 (0.175)	1∙501	-0.545 - 0.143
*Widowed/divorced/separated*	-0.390 (0.253)	0∙820	-0.896 - 0.099
*Never-in-union*	0.406 (0.321)	0∙677	-0.224 - 1.037
**Religion - Mother**			
*Christian (ref)*			
*Islam*	-0.241 (0.182)	0∙786	-0.602 - 0.114
*Traditional*	**-0.844 (0.321)**	**0∙430**	**-1.495 - -0.233**
*Other*	-0.401 (0.272)	0∙670	-0.949 - 0.122
*No religion*	0.033 (0.455)	1∙034	-0.873 - 0.916
**Highest level of Education - Mother**			
*No formal education (ref)*			
*Primary*	-0.090 (0.163)	0∙914	-0.412 - 0.230
*Secondary or High School*	0.044 (0.169)	1∙045	-0.287 - 0.376
*Post-secondary*	0.137 (0.430)	1∙147	-0.691 - 0.997
**Employment status - Mother**			
*Unemployed (ref)*			
*Employed*	**0.396 (0.178)**	**1∙486**	**0.049 - 0.748**
**Health Insurance status**			
*Uninsured (ref)*			
*Insured*	**0.890 (0.189)**	**2∙435**	**0.519 - 1.265**
**Sex of Household head**			
*Male (ref)*			
*Female*	-0.083 (0.147)	0∙920	-0.373 - 0.205
**Household wealth**			
*Poorest (ref)*			
*Poorer*	0.339 (0.174)	1∙404	-0.002 - 0.682
*Middle*	**0.764 (0.191)**	**2∙147**	**0.391 - 1.141**
*Rich*	**0.790 (0.235)**	**2∙203**	**0.328 - 1.253**
*Richest*	**1.112 (0.295)**	**3∙040**	**0.535 - 1.694**
**DHS Period**			
*1993 GDHS (ref)*			
*1998 GDHS*	-0.460 (0.263)	0∙631	-0.976 - 0.059
*2003 GDHS*	0.280 (0.260)	1∙323	-0.226 - 0.794
*2008 GHDS*	0.001 (0.288)	1∙001	-0.561 - 0.568
*2014 GDHS*	-0.187 (0.307)	0∙829	-0.788 - 0.417
**Random Effect**			
Spatial Effect - structured (ICAR model)	6.330 (3.898)		1.707 - 16.407
Spatial Effect - unstructured (IID model)	1851.888 (1831.866)		129.695 - 6687.717
**Model Diagnostics**			
*WAIC*	1905.08		
*DIC*	1902.59		
*Effective Number of parameters (WAIC)*	42.36		
*Marginal log-Likelihood*	-1102.10		
*Variance explained by spatial structure*	0.010		
Mean = Posterior means, SD = Standard deviation, MOR = Mean Odds Ratio, and CI = Confidence Interval			

**Fig. 1:**
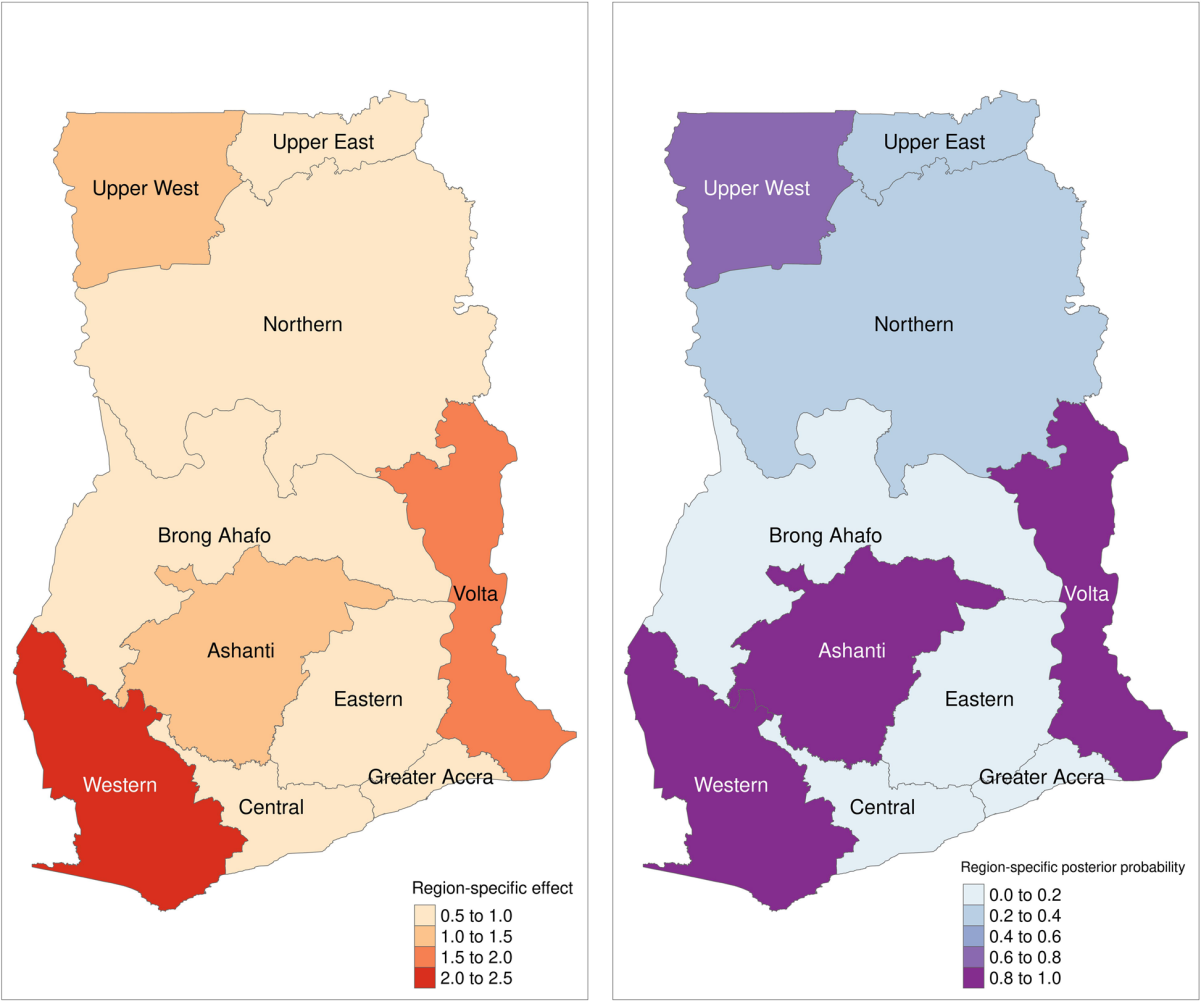
Spatial effects and the exceedance probability of using medical care. Generated with R software using the tmap (version 3.3-2) and tmaptools (version 3.1-1) packages

Source: Authors.

Figure 1 shows the result of the spatial effects or region-specific odds ratio for using medical care for ARI symptoms among children and their probability of exceeding 1. The region-specific effect shows that mothers in the Western, Ashanti, Upper West, and Volta regions have stronger positive effects or increased odds ratio of using a medical treatment for ARI symptoms among children under age 5 years. The opposite effect was observed for mothers in the Upper East, Northern, Brong-Ahafo, Eastern, Central, and Greater Accra regions of the country. Likewise, the figure also reveals the probability that the odds ratio of using medical care for ARI symptoms exceeds 1 is higher for caregivers in the Western, Ashanti, Upper West, and Volta regions.

## Discussions

We found that less than half (43 %) of mothers sought treatment for their children with the onset of ARI symptoms. The majority of mothers in our study– approximately 57 % – did not seek any form of treatment for children with ARI symptoms. Studies in Kenya, Egypt, Tanzania, and Ethiopia [7, 8, 10] found that some mothers visited public health centers when the illness of the children got worse. So our finding may be partly explained by some mothers/caregivers possibly having avoided seeking care unless their child’s symptoms got worse. Delays in seeking health care may have also been due to an inability to pay the cost of such a visit. A study in Ecuador suggests that the lack of money for medicines was an obstacle to seeking timely health care amongst female caregivers [42].

 In both multinomial and bayesian hierarchical logistic regression models, child age, maternal employment status, and maternal health insurance status were identified as determinants of health care seeking. The GDHS period was statistically significant in the multinomial model only. Maternal religion, was also statistically significant in the bayesian hierarchical model. Seeking medical care for ARI symptoms was significantly associated with child age, maternal employment status, household wealth, and national health insurance status. On the other hand, seeking self-care or a traditional healer was significantly associated with maternal education and the GDHS period.

Compared to children aged 4 years, those aged 1 year or less were more likely to have received medical care. This may be partly explained by these mothers having possibly already taken their children to medical centers for post-natal clinics and immunizations during this period. This finding is consistent with those from a study in Tanzania, which found children aged 2 years and older to be less associated with medical care-seeking but rather associated with receiving no care or receiving care at home [9].

The findings suggested that maternal religion played a key role in healthcare-seeking. Mothers who were adherents to traditional religion were significantly associated with a lesser likelihood of seeking medical care compared to their Christian counterparts. Over 70 % of mothers belonging to traditional religion did not seek any form of treatment with the onset of ARI symptoms.

Maternal employment status had a positive relationship with seeking medical care. A mother was more likely to seek medical care for her child’s ARI if she had been employed. Employment is crucial in this regard, as it means that mothers can earn income to support household expenditure. Access to health centers may involve traveling some distance thereby requiring some form of transportation. Incomes accrued through a mother’s employment would, therefore, provide for the cost associated with accessing and using these services. This idea is supported by a study in Kenya, in which inadequate finances were associated with failure to seek health care outside the home [10]. Along similar lines, we also identified household wealth as a significant determinant of medical care. Mothers or caregivers of children living in households that belonged to the middle wealth quintile or higher were more likely to seek medical care, compared to others who belonging to lower quintiles. This finding is consistent with studies in Kenya, Tanzania, Ethiopia, Ecuador and Mongolia [6, 8, 42, 43], where significant relationships were found between household wealth and health care-seeking.

Maternal health insurance registration status was identified as a statistically significant determinant of health care-seeking. Mothers were more likely to seek medical care for ARI symptoms if they had registered with the National Health Insurance Scheme (NHIS). Given that our study population was largely rural (73 %) and largely uninsured (76 %), our findings emphasize the important that the NHIS plays in promoting maternal health care-seeking amongst the rural population in Ghana. Another study has also suggested that mothers whose education was primary school or less and who were from poor households were significantly less likely to be among persons insured under the NHIS [14].

We found lower educational attainment to also be significantly associated with seeking self-care or traditional healing, whereas higher educational attainment was associated with seeking medical care. This finding corroborates earlier studies in Kenya, Tanzania and Ethiopia [6, 8, 9], which suggested that higher educational attainment may have accounted for increased knowledge of symptoms and seeking medical attention at a health facility outside the home. The GDHS period was significantly associated with seeking self-care or traditional healers, which may be partly explained by access to medical care facilities, services, and health insurance having been lower within the GDHS periods 1993 to 2014 [15, 19, 22].

We observed that mothers living in the Upper East, Northern, Brong-Ahafo, Eastern, Central, and Greater Accra regions had lesser odds of seeking medical care for their children. Also, mothers in rural areas were less likely to seek medical care compared to their urban counterparts. A disparity in the spatial distribution of medical health facilities in urban areas relative to rural areas in Ghana may have been a contributory factor in this finding [14, 19, 25, 28]. As suggested by a study in Kenya, government hospitals are where medical care is often received, but they may have been relatively far away from where people live and have been costly [6]. Additionally, mothers may have had to travel (walking or paying for transport) longer distances compared to their urban counterparts to seek health care. This is an important finding, given that our study population living in the southern parts of the country had a higher exceedance probability of using medical care relative to those in the northern parts.

Our study should be viewed in light of the following limitations. First, our operational definition of medical care excluded ‘Pharmacy or drugstore’ but some pharmacies or drug stores might, nevertheless, have provided medical care to our study population. Accordingly, our findings should be interpreted in that context. Second, mothers provided answers based on a two-week recall of the health status of their children, and some of their answers may have been subject to recall bias. Third, a longitudinal study design may have afforded greater insight into the contributions of determinants across the wet and dry seasons in Ghana. Fourth, the cross-sectional design affords a snapshot of maternal healthcare-seeking and does not afford an interpretation of causality. Fifth, the mother’s proximity to health centers and the time taken to access health services were not measured quantitatively. Furthermore, our study does not capture the quantity and quality of health care infrastructure and health care delivery over time, as this was outside the scope of the study. Despite these limitations, our study has some strengths. It represents the first attempt to pull together GDHS data sets to assess determinants of health care-seeking for children with ARI symptoms. It therefore contributes to the growing body of literature on the determinants of health care seeking for childhood ARI amongst mothers/caregivers.

## Conclusions

Our study found that the type of health care sought for symptoms of childhood ARI was determined by maternal socioeconomic and household characteristics. We identified the child’s age, maternal employment status, household wealth, maternal health insurance status and the GDHS period as significant determinants of choosing medical care. The choice for self-care or traditional healer was significantly associated with maternal education status and the GDHS period. Our findings suggest that interventions aimed at improving maternal socioeconomic and household conditions may improve medical care-seeking for childhood ARI symptoms. Policies aimed at improving maternal employment opportunities, improving maternal education on the symptoms and management of childhood ARI and policies geared towards encouraging mothers to enrol in the National Health Insurance Scheme may also yield positive effects for medical healthcare-seeking. Findings from our Bayesian spatial model suggest that there is a need to reduce regional disparities in socioeconomic indicators and the distribution of health care resources. Future studies could shed more light on the roles that sociocultural, religious, and physical disability factors play in health care-seeking for childhood ARI symptoms. In general, the government and its stakeholders should strengthen efforts at improving the national socioeconomic and health systems to overcome the problem of acute respiratory infection in children.

## Data Availability

The datasets which were analyzed during the current study are publicly available at: https://dhsprogram.com/data/available-datasets.cfm.

## Abbreviations

ARI: Acute Respiratory Infection
BYM: Besag-York-Mollie
CHPS: Community- based Health Planning and Services
EA: Enumeration area
GDHS: Ghana Demographic and Health Survey
GHS: Ghana Health Service
MOR: Mean Odds Ratio
NHIS: National Health Insurance Scheme
RRR: Relative risk ratio
SRS: Simple random sampling
